# Early versus delayed treatment of lateral condylar fracture of the humerus with > 2 mm displacement in children: a retrospective study

**DOI:** 10.1186/s13018-023-03619-6

**Published:** 2023-02-24

**Authors:** Biao Wang, Rongxuan Gao, Zhenwei Li, Zhanhao Guo, Zejuan Ji, Weili Li, Keming Sun

**Affiliations:** 1 Department of Orthopaedics, Children’s Hospital Affiliated to Zhengzhou University, Henan Children’s Hospital, Zhengzhou Children’s Hospital, Zhengzhou, Henan China; 2 Department of Orthopaedics, Beijing Children’s Hospital, Capital Medical University, National Center for Children’s Health, Beijing, China

**Keywords:** Lateral condylar fracture, Humerus, Delayed surgery, Incision length, Pediatric

## Abstract

**Background:**

The purpose of this study was to investigate the clinical and functional outcomes of early versus delayed treatment of pediatric lateral condylar fractures of the humerus with a displacement greater than 2 mm.

**Methods:**

Sixty-seven children treated surgically at our hospital from March 2016 to September 2021 for lateral condylar fracture of the humerus with displacement > 2 mm were retrospectively analyzed. The children were divided into two groups where early surgery consisted of patients being operated on within 24-h post-injury (*n* = 36) and delayed surgery consisted of children operated after 24-h post-injury (*n* = 31). Clinical and functional results were compared between the two groups.

**Results:**

There were no significant differences between the two groups in terms of operation time, blood loss and incidences of perioperative complications. However, mean length of incision was significantly greater (*P* < 0.0001) in the delayed treatment group (5.68 ± 1.08 cm) compared to the early treatment group (3.89 ± 0.82 cm). No differences were found in functional outcomes, consisting of the Baumann angle of the affected limb, the carrying angle, Mayo Elbow Performance Score, and Flynn’s criteria at final follow-up.

**Conclusions:**

Delay in surgery for more than 24 h after injury does not influence the clinical and functional results for lateral condylar fracture of the humerus with displacement > 2 mm in children. However, delayed open reduction and pinning may increase the incision length possibly due to increased edema.

## Background

Lateral condylar fracture of the humerus (LCFH) is one of the most common fractures in children and the most common elbow fracture that involves the growth plate [1]. These fractures of the distal humerus can be problematic in terms of diagnosis, treatment and complications [2]. Shabir et al. [3] hypothesized that the incidence of functional loss in the range of motion (ROM) of the elbow is much greater with LCFH because the fracture line extends into the articular surface. Treatment for displaced LCFH with open reduction and internal fixation (ORIF) is accepted worldwide as the primary method of treatment [4, 5].

The effect of early versus delayed treatment has been a topic of interest for various fracture types [6–9]. It is possible that early operative treatment for LCFH may prevent complications such as infection, malunion and nonunion [10]. However, few studies in the literature comparing early and delayed surgery for displaced fractures of the lateral condyle of the humerus have been reported. In this retrospective study, we compared the results of early ORIF and delayed ORIF in treating closed LCHF with displacements greater than 2 mm.

## Methods

### Ethics

This study was approved by the Ethics Review Committee of the hospital (No. 2022-K-092, November 11, 2022). Informed consent was waived as this was a retrospective study. The study was conducted following the ethical principles of the Declaration of Helsinki.

### Participants

Data from 67 pediatric patients with LCFH were identified through our hospital database from March 2016 to September 2021. Patients were divided into an early operation group (*n* = 36) that received surgical treatment within 24 h of injury and a late operation group (*n* = 31) consisting of patients who received surgical treatment between 24–72 h of injury.

The inclusion criteria consisted of patients with unilateral closed Song type IV and V LCFH [11], aged 1–14 years old, received ORIF treatment, had a displaced fracture > 2 mm and a follow-up period up to at least one year. The displacements of the intra-articular fractures were measured through preoperative radiographs of each patient. The exclusion criteria consisted of patients with open fractures, pathological fractures, combined fractures affecting the target elbow joint rehabilitation, compartment syndrome, severe neurovascular insufficiency, and lack of follow-up information. To minimize the confounding bias, patients surgically treated after 72 h from time of injury to surgical treatment were also excluded.

### Surgical technique

Open reduction was conducted in the supine position under general anesthesia, and an approximately 4–5-cm-long incision was made on the lateral side of the elbow joint. After blunt-scissor dissection of the subcutaneous tissue, the anterior side of the lateral condyle was inspected where articular fracture line was confirmed. Open reduction was then conducted. Following direct confirmation of anatomical reduction, 2–3 Kirschner wires (K-wires) (1.5 or 2.0 mm) were inserted from the lateral condyle under direct visualization of the articular surface. During the surgery, meticulous care was taken to preserve the soft tissue attachment of the lateral condylar fragment posteriorly. After confirming pin and wire configurations fluoroscopically, K-wires were unburied and the region was covered with iodoform gauze. Post-operation, the arm was immobilized in a long-arm cast in a functional position for 4–6 weeks.

### Postoperative care and follow-up

Intravenous prophylactic antibiotics (cefuroxime sodium, 30 mg/kg) were routinely administered 30 min pre-operation and once postoperatively. Follow-up appointments with clinical and radiographic evaluation were scheduled regularly every 2 weeks after surgery until bone union was confirmed. If the callus bridging the fracture gap was confirmed in X-ray film 4–6 weeks post-operation, the cast and K-wires were removed in the outpatient clinic. Functional rehabilitation exercises were then initiated. Three months and one year after operation, patients were required to go to the outpatient clinic for follow-up.

### Data collection and outcome indicators

Data collected from the patients included age, sex, date and time of admission, operating conditions, perioperative complications, and results of functional rehabilitation. Operating conditions included operation time, blood loss and incision length. Complications recorded consisted of superficial infections, nonunions, malunions, lateral condylar prominence, cubitus varus deformity, nerve damage and osteonecrosis of the lateral humeral condyle. Measurements for functional scores included:

1. The Baumann angle of the affected limb and the contralateral healthy limb of the two groups were measured at the last follow-up. The Baumann angle consists of the angle between the oblique line passing through the lateral epiphyseal plate and the central axis of the humeral shaft with an average of 72° (64°–81°).
2. Carrying angle of the affected limb and the contralateral healthy limb of the two groups were measured at the last follow-up. The carrying angle consisted of the intersection of the arm axis and the extension line of the forearm axis to form an outwardly open angle, about 165°–170° with a supplementary angle of 15 ± 5°.
3. Mayo Elbow Performance Score (MEPS) [12] was used to assess elbow joint function at final follow-up, which included pain (45 points), elbow stability (10 points), range of motion (20 points) and daily functional tasks (25 points). Higher scores out of 100 indicated better elbow function and categorized into excellent (90–100), good (75–89), fair (60–74), and poor (0–59).
4. Flynn’s criteria [13] were used to evaluate treatment outcome at last follow-up. Measurements were graded as follows: limited elbow flexion and extension < 5°, carrying angle loss < 5° (excellent); limited elbow flexion and extension of 6°–10°, carrying angle loss of 6°–10° (good); limited elbow flexion and extension of 11°–15°, carrying angle loss of 11°–15° (fair); elbow flexion and extension limited > 15°, carrying angle loss > 15° (poor).

### Statistical analysis

Lognormal distribution of the data was assessed based on the D’Agostino and Pearson test. Student’s *t* test, Mann–Whitney *U* test and chi-square test or Fisher’s exact test was used to compare parametric numerical data, nonparametric numerical data and categorical data, respectively. Statistical analysis was conducted using GraphPad Prism 9.

## Results

### Patient characteristics

Patient demographics and perioperative parameters are shown in Table 1. The early operation group consisted of 30 males and 6 females with a mean age of 4.6 ± 2.2 years old (range: 2–11 years old), mean time from injury to operation of 13.1 ± 4.1 h (range, 6–23 h). The delayed operation group consisted of 21 males and 10 females with a mean age of 4.1 ± 1.9 years old (range: 1.1–8 years old), mean time from injury to operation of 43.8 ± 16.6 h (range, 25–71 h). There were no significant differences between gender and age among the two groups (*P* > 0.05).

**Table 1:** Patient characteristics and perioperative parameters

	Early treatment group (*n* = 36)	Delayed treatment group (*n* = 31)	*P* value
Age (years)	4.6 ± 2.2	4.1 ± 1.9	0.3381*
Sex (male:female)	30:6	21:10	0.1356^†^
Time from injury to surgery (hours)	13.1 ± 4.1	43.8 ± 16.6	< 0.0001**
Song classification			0.5805^††^
Type IV	27	25	
Type V	9	6	
Operation length (minutes)	68.9 ± 21.0	72.3 ± 26.0	0.5599*
Blood loss (mL)	9.7 ± 1.2 mL	10.0 ± 1.2 mL	0.2570*
Incision length (cm)	3.89 ± 0.82	5.68 ± 1.08	< 0.0001*
Complications			
Superficial infection	1	2	> 0.9999^†^
Lateral condylar prominence	8	7	0.9720^††^
Carrying angle loss > 5°	6	6	0.7748^††^

*Student’s *t* test; **Mann–Whitney *U* test; ^†^Fisher’s exact test; ^††^chi-square test

### Perioperative parameters

All patients underwent open reduction and internal fixation with K-wire. The mean duration of surgery was 68.9 ± 21.0 min (range: 40–120 min) for the early group and 72.3 ± 26.0 min (range: 40–145 min) for the delayed group (*P* = 0.5599). Patients in the early group had a mean blood loss of 9.7 ± 1.2 mL (range: 8–12 mL) versus 10.0 ± 1.2 mL (range: 8–12 ml) in the delayed group (*P* = 0.2570). However, the differences in incision length were statistically significant. The mean incision length was 3.89 ± 0.82 in the early treatment group and 5.68 ± 1.08 in the delayed group. There was no statistical difference between these two groups regarding superficial infections, cubitus varus, and lateral prominence (*P* > 0.05). There were no incidences of nerve injury, malunion, nonunion or avascular necrosis.

### Functional outcomes

Functional outcomes are displayed in Table 2. Baumann angle for the early treatment group was 70.50 ± 5.63° (range: 61–81°) compared to 71.42 ± 5.97° (range 62–83°) in the delayed treatment group. Carrying angle was 9.71 ± 2.87° (range: 3–15°) in the early treatment group and 9.68 ± 3.90° (range: 1–16°) in the delayed group. MEPS was 95.42 ± 5.53 (range: 80–100) in the early treatment group and 94.84 ± 5.40 (range: 80–100) in the delayed group. Based on Flynn’s criteria, early treatment had 28 excellent, 5 good and 3 fair outcomes whereas the delayed treatment group had 24 excellent, 5 good, 1 fair and 1 poor outcome. No significant differences were found between any of the functional outcomes with *P* values all greater than 0.05.

**Table 2:** Comparison of functional outcomes between early and delayed treatment for lateral condylar fracture of the humerus

	Early treatment group (*n* = 36)	Delayed treatment group (*n* = 31)	*P* value
Baumann angle (°)	70.50 ± 5.63	71.42 ± 5.97	0.5192*
Carrying angle (°)	9.71 ± 2.87	9.68 ± 3.90	0.7287**
MEPS	95.42 ± 5.53	94.84 ± 5.40	0.5437**
Flynn’s criteria			> 0.9999^†^
Excellent	28	24	
Good	5	5	
Fair	3	1	
Poor	0	1	

*Student’s *t* test; **Mann–Whitney *U* test; ^†^Fisher’s exact test with excellent/good in one category and fair/poor in the other category

## Discussion

The impact of delaying surgical treatments for various fractures has always been a focus of clinical research. Due to various factors, such as a missed diagnosis, extended transportation times, or issues with insurance, cases of pediatric fractures do not always receive timely surgical treatment, which can be a more critical issue in developing countries. On the other hand, daytime-dedicated orthopedic trauma rooms may be a viable option to improve patient flow and cost savings. An increasing number of hospitals are developing orthopedic trauma rooms and finding that complication rates do not increase in either the pediatric or adult population [6].

Studies in the literature on LCFH have primarily focused on the effect of surgical treatment delays of over three weeks, to which three-week-old fractures are considered old fractures. Mulpruek et al. [14] reported that surgical intervention for neglected and displaced LCFH carries a potential risk of avascular necrosis, infection, stiffness of the joint, and growth disturbance of the distal humerus. On the other hand, satisfactory outcomes have been reported in other studies [15–17], especially in patients with an early-delayed presentation. Li et al. [10] retrospectively studied 43 lateral LCFH where 17 of their patients were treated with K-wires and 26 patients were treated with biodegradable pins. The authors hypothesized that open reduction and internal fixation for LCFH with an early-delayed presentation produced satisfactory outcomes. They found that biodegradable pins were a good alternative to K-wires with comparable clinical outcomes.

This study is the first report in the literature that examined the delayed treatment of fresh LCFH, using a 24-h threshold. At present, there is no consensus on a specific, standardized time threshold on early emergency surgeries of pediatric fractures in the literature. Most researchers set an early surgical time of 8 h, 12 h or 24 h [8, 9, 18–20]]. If 12 h was taken as the standard time for early surgery in our study, less than 30% of the patients would have been eligible for the early surgery group. Therefore, in this study, the indicator for early emergency operation from injury to surgical treatment was set to a time period of less than 24 h, and a delayed surgery was established as a wait period of greater than or equal to 24 h. Delayed hospital visits or delayed surgical treatment of LCFH is common. Due to a variety of reasons, many patients with LCFH often initially receive closed reduction and cast immobilization or direct cast immobilization. Delayed treatment is also prevalent in cases where the patient is transferred from another hospital. As the hospital in this study is a provincial pediatric medical center, pediatric patients with fractures are often transferred from lower tier hospitals situated over 100 km away in order to receive surgical treatment at our hospital. Thus, the time from injury to surgery in our hospital often exceeds 24 h. Consequently, we believe that using 24 h as an indicator for early emergency surgery might have more practical clinical significance.

The results of our study showed that perioperative complications, length of surgery, and functional outcomes were not significantly different in patients treated within 24 h compared to patients treated between 24–72 h. Satisfactory clinical outcomes were achieved in both groups with a low rate of complication. However, there was a significant difference in surgical incision length between the two groups where incision length was longer in the delayed treatment group compared to the early treatment group. One possible reason for this difference is that it is easier to achieve a suitable reduction if the surgery was performed earlier before the development of swelling. Another reason for the increased incision length could be due to the surgical practices of our department. For open reduction, especially for fractures with severe swelling of soft tissue, additional care and attention is dedicated to avoid stripping the soft tissue at both ends of the fracture. Instead, the surgical decision might be made to appropriately enlarge the incision to aid reduction.

Limitations of this study are that it was a retrospective, cohort study. However, due to ethical considerations, a randomized study would not be possible. Another limitation is that only two surgeons were included for the cases in this study. Variation in surgical techniques and skill level could possibly affect perioperative and clinical outcomes. The presence of many possible confounding factors such as Song type, which can influence the results of our study, was a concern. For these reasons, the correlations reported in our study must be interpreted with caution.

## Conclusion

In summary, the results of this study suggest that patients with a 24–72-h delay in surgical treatment for LCFH have the same outcomes as patients who are treated within 24 h of injury. However, surgical incision sizes could be longer in delayed treatments due to increased edema and swelling, which would increase the difficulty of achieving a suitable reduction.

## Data Availability

The datasets used and/or analyzed for the current study are available from the corresponding author on reasonable request.
